# Covidence and Rayyan

**DOI:** 10.5195/jmla.2018.513

**Published:** 2018-10-01

**Authors:** Liz Kellermeyer, Ben Harnke, Shandra Knight

**Affiliations:** Biomedical Research Librarian, Library and Knowledge Services, National Jewish Health, Denver, CO; Education and Reference Librarian, Health Sciences Library, University of Colorado Anschutz Medical Campus, Aurora, CO; Director, Library and Knowledge Services, National Jewish Health, Denver, CO

## INTRODUCTION

Increasingly important in health care settings, systematic reviews (SRs) aim to identify, evaluate, and summarize the relevant studies of a health-related issue. Librarians respond to the need for SRs with expert search services, project management, and training, making them well positioned to help teams establish best practices during the review process.

To minimize bias and present reliable evidence, SRs adhere to a design based on structured and reproducible methods, which include steps to search for relevant studies, extract data, assess the quality of the data, and then analyze and present the results. Several groups, such as Cochrane and the Institute of Medicine, have set forth guidelines for conducting systematic reviews [1, 2]. Additionally, Preferred Reporting Items for Systematic Reviews and Meta-Analyses (PRISMA) formatting is commonly adopted to summarize and report SR findings.

Numerous tools are available that have been designed to help with one or more steps in the SR process. The aim of this review is to compare two SR tools, Covidence and Rayyan, and examine the ways in which they support SR methodology and reporting standards. Covidence is free for Cochrane authors and fee-based for others. It was developed specifically to guide reviewers through a prescribed SR workflow. Rayyan is a completely free tool developed to expedite the SR process by easing citation sharing and allowing comparison of decisions to include or exclude.

Before starting a review project, participants should be clear about the degree to which and the means by which they will adhere to best practices. While methodological quality is distinct from reporting quality, one cannot evaluate how well the SR was conducted with incomplete documentation [3]. Both are needed for an effective summary of the evidence. Transparency, reproducibility, and publisher-defined requirements should all inform the selection of an SR tool.

## COVIDENCE

Covidence was developed by an Australian not-for-profit company. In 2015, Cochrane initiated a partnership with Covidence that made it the standard production platform for Cochrane reviews. While an instance of Covidence (a single review) is free for Cochrane authors, others receive 1 free trial with a maximum of 2 reviewers and then face fees. One instance costs $240, which includes an unlimited number of reviewers. Pricing schedules vary and include options for institutional subscriptions and bulk purchasing. Covidence mirrors the multiphase review process, including data extraction, directly in its design. Citations neatly progress through each stage based on votes received. At every stage, reviewers can explicitly assign voting roles, including tie breaking, while maintaining blinding, which helps to minimize bias.

## RAYYAN

Rayyan was developed through the Qatar Computing Research Institute, funded by the Qatar Foundation, a nonprofit that supports education, science, research, and community development initiatives in Qatar. It is completely web-based, with offline compatibility through its app. Users are able to initiate and/or participate in an unlimited number of reviews. As opposed to Covidence, Rayyan does not easily mirror the multiphase citation review process and is really only designed to aid with the reference screening. It takes a minimalist approach, placing more of the logistical and workflow burden on the users themselves.

## COMPARISON

Table 1 compares the two products. In essence, Covidence protects the integrity of the systematic review process. It lacks flexibility by design in order to minimize the chance that reviewers could do anything to compromise the review process or data reporting. It has built-in capabilities to handle the screening process in a way that reduces duplicate efforts on behalf of the reviewers. For example, if 100 articles need a vote by 2 reviewers and there is a team of 3 reviewers, Covidence will track include/exclude decisions as they are made, removing an article from the review queue if it has met the requirement of receiving two votes. In this way, the review workload can be spread more easily amongst the team. In contrast, Rayyan’s reviewer blinding function can only be turned on or off. If the same team of 3 wants a blinded review of those 100 articles, the articles would need to be manually split up or each reviewer would need to vote on all articles.

**Table 1:**
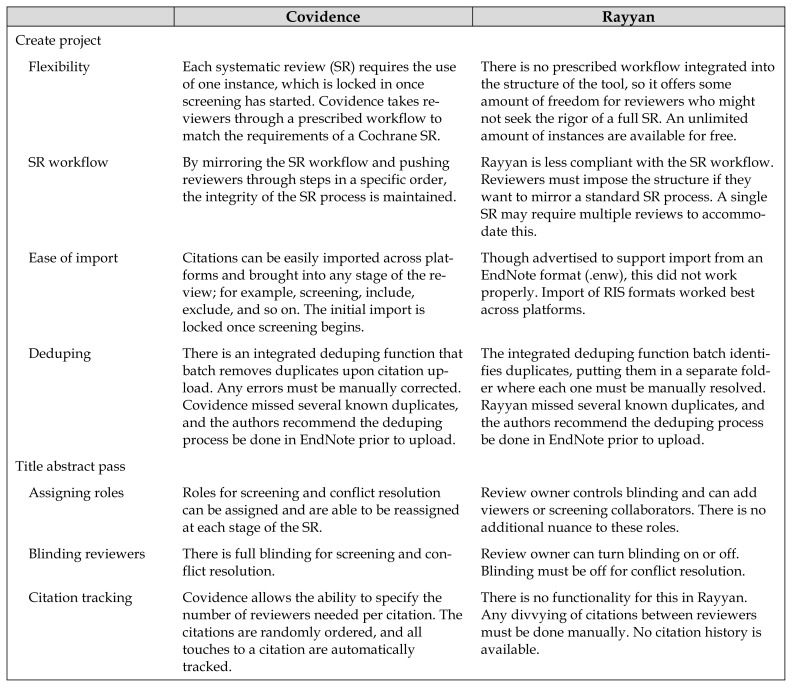
Comparison of key features between Covidence and Rayyan as mirrored through a standard systematic review workflow

	Covidence	Rayyan
Create project		
Flexibility	Each systematic review (SR) requires the use of one instance, which is locked in once screening has started. Covidence takes reviewers through a prescribed workflow to match the requirements of a Cochrane SR.	There is no prescribed workflow integrated into the structure of the tool, so it offers some amount of freedom for reviewers who might not seek the rigor of a full SR. An unlimited amount of instances are available for free.
SR workflow	By mirroring the SR workflow and pushing reviewers through steps in a specific order, the integrity of the SR process is maintained.	Rayyan is less compliant with the SR workflow. Reviewers must impose the structure if they want to mirror a standard SR process. A single SR may require multiple reviews to accommodate this.
Ease of import	Citations can be easily imported across platforms and brought into any stage of the review; for example, screening, include, exclude, and so on. The initial import is locked once screening begins.	Though advertised to support import from an EndNote format (.enw), this did not work properly. Import of RIS formats worked best across platforms.
Deduping	There is an integrated deduping function that batch removes duplicates upon citation upload. Any errors must be manually corrected. Covidence missed several known duplicates, and the authors recommend the deduping process be done in EndNote prior to upload.	The integrated deduping function batch identifies duplicates, putting them in a separate folder where each one must be manually resolved. Rayyan missed several known duplicates, and the authors recommend the deduping process be done in EndNote prior to upload.
Title abstract pass		
Assigning roles	Roles for screening and conflict resolution can be assigned and are able to be reassigned at each stage of the SR.	Review owner controls blinding and can add viewers or screening collaborators. There is no additional nuance to these roles.
Blinding reviewers	There is full blinding for screening and conflict resolution.	Review owner can turn blinding on or off. Blinding must be off for conflict resolution.
Citation tracking	Covidence allows the ability to specify the number of reviewers needed per citation. The citations are randomly ordered, and all touches to a citation are automatically tracked.	There is no functionality for this in Rayyan. Any divvying of citations between reviewers must be done manually. No citation history is available.
Conflict resolution	There is the ability to assign a tie-breaker role, and then the voting history is blinded for this person.	Conflicts must be resolved manually through unblinded consensus. A chat feature is available for this purpose.
Full-text pass		
Bulk full-text import	Bulk portable document format files (PDF) import from EndNote is possible, though the multistep process is arduous.	No bulk import function is available, so each PDF must be attached individually.
Exclusion criteria	Some prepopulated exclusion criteria are suggested. Users can delete unwanted ones or add new ones to create a customized list to choose from. If a citation is excluded at this stage, a single criterion must be selected. If reviewers select different criteria, it is considered a conflict that must be resolved.	Some prepopulated exclusion criteria are suggested. Users can add new ones, but cannot delete, creating a somewhat muddy exclusion list. When excluding a citation, adding a criterion is optional. Multiple exclusion criteria are able to be added to a single citation, which could confuse the SR process.
Conflict resolution	There is the ability to assign a tie-breaker role, and then the voting history is blinded for this person. For excluded citations, agreement must be reached on a single exclusion criterion.	Conflicts must be resolved manually through unblinded consensus. A chat feature is available for this purpose.
Data extraction		
Quality assessment	Reviewers can use Cochrane risk of bias or a custom template.	Unavailable
Data extraction	Robust data extraction is available for meta-analysis.	Unavailable
Reports		
Creating reports	Generates PRISMA flow diagram and can export quality assessment, risk of bias, and study data to RevMan to generate reports.	Unavailable

Resolving conflicts is easier in Covidence, which has an integrated conflict resolution workflow that allows a designated person to be a tie-breaker. Though blinding is possible during initial voting in Rayyan, conflicts can only be resolved by unblinding the decisions, having reviewers discuss, and manually changing votes. A chat feature is available to aid in this process.

Once articles pass into the full-text phase, Covidence requires that reviewers add a single reason when choosing to exclude an article. If different reviewers choose different reasons for exclusion, Covidence will classify it as a conflict that needs to be resolved. This gives each article a single exclude reason, providing tidy data for the final exclude table needed for SR reporting.

In contrast, there is no full-text phase designated in Rayyan’s interface. The best practice for mimicking this phase would be to export the citations that were included in the first pass and import them back into a new Rayyan review with the initial decisions stripped from them. After that, full-text portable document format files (PDFs) could be uploaded individually, and reviewers could proceed as in the first pass. Rayyan allows reviewers to skip adding an exclude reason or to add multiple reasons. While resolving all conflicts at the full-text phase is possible through Rayyan, a workflow would need to be established prior to beginning. Data necessary for the exclude table would need to be manually extracted.

Finally, Covidence offers tools and a workflow for all data extraction that would be necessary for the final SR reporting. It produces a PRISMA flow diagram and is able to export additional information to RevMan, a software program that can produce a meta-analysis and graphic representation of the data. Rayyan does not support any additional phases of the SR workflow past the screening. Covidence is well-suited for more rigorous SRs, where methodology must be adhered to and documented at each stage. Though workflow and features can feel clunky, Rayyan offers a nice structure for the initial screening process and works as a viable upgrade from a workflow that uses only EndNote and/or Excel.
